# Evaluation of Antithrombotic Activity of Thrombin DNA Aptamers by a Murine Thrombosis Model

**DOI:** 10.1371/journal.pone.0107113

**Published:** 2014-09-05

**Authors:** Elena Zavyalova, Nadezhda Samoylenkova, Alexander Revishchin, Andrey Golovin, Galina Pavlova, Alexey Kopylov

**Affiliations:** 1 Chemistry Department of Lomonosov Moscow State University, Moscow, Russian Federation; 2 ‘APTO-PHARM’ LTD, Moscow, Russian Federation; 3 Institute of Gene Biology of Russian Academy of Sciences, Moscow, Russian Federation; 4 Department of Bioengineering and Bioinformatics of Lomonosov Moscow State University, Moscow, Russian Federation; Beckman Research Institute of the City of Hope, United States of America

## Abstract

Aptamers are nucleic acid based molecular recognition elements with a high potential for the theranostics. Some of the aptamers are under development for therapeutic applications as promising antithrombotic agents; and G-quadruplex DNA aptamers, which directly inhibit the thrombin activity, are among them. RA-36, the 31-meric DNA aptamer, consists of two thrombin binding pharmacophores joined with the thymine linker. It has been shown earlier that RA-36 directly inhibits thrombin in the reaction of fibrinogen hydrolysis, and also it inhibits plasma and blood coagulation. Studies of both inhibitory and anticoagulation effects had indicated rather high species specificity of the aptamer. Further R&D of RA-36 requires exploring its efficiency *in vivo*. Therefore the development of a robust and adequate animal model for effective physiological studies of aptamers is in high current demand. This work is devoted to *in vivo* study of the antithrombotic effect of RA-36 aptamer. A murine model of thrombosis has been applied to reveal a lag and even prevention of thrombus formation when RA-36 was intravenous bolus injected in high doses of 1.4–7.1 µmol/kg (14–70 mg/kg). A comparative study of RA-36 aptamer and bivalirudin reveals that both direct thrombin inhibitors have similar antithrombotic effects for the murine model of thrombosis; though *in vitro* bivalirudin has anticoagulation activity several times higher compared to RA-36. The results indicate that both RA-36 aptamer and bivalirudin are direct thrombin inhibitors of different potency, but possible interactions of the thrombin-inhibitor complex with other components of blood coagulation cascade level the physiological effects for both inhibitors.

## Introduction

The hemostasis is responsible for keeping the blood in a liquid state that is to balance preventing the bleedings with thrombus formation and dissolving the unwanted thrombi. Two main mechanisms maintain hemostasis: aggregation of platelets and formation of the fibrin fibers [1], [2]. Therefore two classes of the antithrombotic substances are used to prevent thrombus formation: anti-aggregants (antiplatelet agents) and anti-coagulants (inhibitors of the blood coagulation cascade), respectively. Drugs of both classes are widely used in the therapy of thrombosis [3], [4]; though research and development of new safe drugs with predictable activity are in great demand of modern medicinal chemistry.

The thrombin is a conventional target for searching new anticoagulants. The thrombin is a serine-type peptidase which is generated in the blood as a result of initiating of the coagulation cascade. The major substrate of the thrombin is fibrinogen which is hydrolyzed into fibrin, the latter forms a mesh for the thrombus scaffold [5]. The direct thrombin inhibitors belong to different classes of chemicals and biologics: aromatic chemicals, peptidomimetics, peptides, proteins, polysaccharides, and oligonucleotides [6], [7]. The later ones are both DNA aptamers and RNA aptamers [8].

Nucleic acid based aptamers are a promising class of molecular recognition elements that have a high affinity and selectivity for a variety of targets ranging from ions up to the living cells. Aptamers are oligonucleotides (DNA or RNA) with a specific three dimensional structure that specifically interacts (recognizes) the target. A very unique feature of the aptamers is a possibility to have a rational antidote, a complementary oligonucleotide, which destroys a specific 3D structure of the aptamer by making a double helix, and therefore eliminates the aptamer interactions with the target [9], [10].

Up till now a number of the coagulation factors have become a target for the aptamer selection: factor IIa (thrombin) [11]–[14], factor VII [15], factor IX [16]–[18], factor X [19], factor XII [20], tissue factor pathway inhibitor (TFPI) [21], protein C [22], and von Willebrand factor [23]–[26].

This study has focused on the antithrombotic activity of RA-36 aptamer, DNA 31-mer to thrombin, which has been described recently. RA-36 aptamer has two covalently linked guanine quadruplexes, each represents the thrombin-binding pharmacophore. Previously the anticoagulant activity of RA-36 aptamer has been studied in both enzymatic and coagulation tests [27]–[30]. This study describes *in vivo* antithrombotic activity of RA-36 aptamer in the animal model. For that purpose a murine thrombosis model has been adapted. It turned out that the antithrombotic effect of RA-36 aptamer is similar to that one of bivalirudin [31], the 20-mer peptide anticoagulant, which is already commercially available as a drug.

## Materials and Methods

Inorganic salts and Tris were purchased from MP Biomedicals (France). Recombinant human thrombin with a specific activity of 3.6 kIU mg^−1^, and murine thrombin with a specific activity of 3.8 kIU mg^−1^ were from HTI, USA; human plasma fibrinogen was from Calbiochem, Germany; bivalirudin trifluoroacetate was from Selleck Chemicals, USA. DNA oligonucleotide RA-36 (GGTTGGTGTGGTTGGTGGTTGGTGTGGTTGG) was synthesized by ‘APTO-PHARM’ LTD, Russian Federation. Standard human plasma, thrombin time reagent, and prothrombin time reagent for coagulation tests were from Siemens, Germany; murine citrated plasma was purchased from Sigma-Aldrich, USA.

### Aptamer Preformation

Potassium chloride was used to stabilize the G-quadruplex structure of the aptamer. Generally RA-36 aptamer (400 nM, 50 µM, and 5 mM) in 10 mM aqueous solution of KCl was heated at 95°C during 5 min, and then cooled at room temperature to facilitate an assembly of the G-quadruplex structure.

### Turbidimetric Assay

The thrombin inhibiting experiments were performed in a buffer mimicking salt composition of the plasma: 20 mM Tris-acetate, pH 7.4, 0.14 M NaCl, 5 mM KCl, 1 mM MgCl_2_, 1 mM CaCl_2_, at 37°C. Fibrinogen concentration was varied from 0.5 to 2.0 µM, and the thrombin addition (final concentration 2 nM) was taken as a reference point. The sample turbidity was measured by the spectrophotometer WPA Biowave II+ (Biochrom, UK). Each experiment was performed at least three times.

Either preformed RA-36 aptamer (final concentration in 2–36 nM range) or bivalirudin (final concentration in 0.5–8 nM range) were added into the assay straight before the thrombin.

The inhibition coefficient was calculated according to the equation (1):
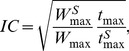
(1)where 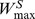 and 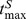 the parameters of the turbidimetric curve for the standard sample, and *IC* is the inhibition coefficient which means a decreasing of the active thrombin concentration by the inhibitor. The inhibition types and constants were determined according to Zavyalova *et al.* [32]. The equation for complete non-competitive inhibition type described here is as follows:
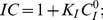
(2)where 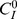 is the inhibitor concentration, and 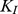 is apparent association constant for the thrombin-inhibitor complex.

### Coagulation Tests

Both the thrombin time and the prothrombin time tests for standard human plasma were performed using the coagulometer CA-50 (Sysmex, Japan) according to the reagent manufacture's guidance. Experiments on murine plasma were conducted similarly. To estimate the anticoagulant activity of either RA-36 aptamer or bivalirudin, the inhibitor was added to the plasma sample prior the coagulation experiment. Each experiment was performed at least twice.

### The murine model of thrombosis

The model is a modification of the reported previously murine model of arterial thrombosis with electric injury [33]. All procedures were conducted in accordance with the standards set forth in the EU Directive 2010/63/EU. All animal care and experimental procedures were approved by the Ethics Committee of Moscow State University (Permit Number 24-01). A total of 42 male C57Bl/6 mice (12 weeks age and 25–31 g) used in the experiments were supplied from the Experimental Animals Unit of Blokhin Russian Cancer Research Center. They were maintained in a standard laboratory animal facility with free access to feed, water, a reverse 12 h∶12 h light: dark cycle and were acclimatized to these conditions for at least two weeks before the start of the experiments. Mice were anaesthetized with ketamine (80 mg·kg^−1^) and xylazine (20 mg·kg^−1^). Anaesthesia was monitored using pedal reflex. Lidocaine was used for local anaesthesia at the site of surgery. Carotid artery and jugular vein were denuded. Carotid artery was isolated from surrounding tissues with a piece of polyethylene. 100 µl of the sample of physiological solution (0.14 M NaCl), or aptamer RA-36 (14–70 mg kg^−1^ dose), or bivalirudin (3.75–7.5 mg kg^−1^ dose) was bolus injected into the jugular vein. Injected samples were encoded to provide blinded experiments. A thin steel needle (the injection needle of the tuberculin syringe) was contacted with the denuded carotid artery under control of stereomicroscope. The second needle was introduced subcutaneously into the hip of the mouse. Both needles were used as the electrodes connected to battery of the constant current (voltage - 3 V, amperage - 200–250 µA) for 60–120 sec after the sample injection. Current damages endothelium of the vessel; and thrombus occurs near the site of the contact of the needle with artery. The animal was placed on the table of Olympus microscope, and the video was made with the digital camera Nikon D5100 during 3–24 min after the sample injection. Each experiment was performed at least for six animals. To minimize suffering, mice were euthanized by one intramuscular injection of 150 mg kg^−1^ ketamine.

The video was analyzed using software Image Tool, which captures discrete frames with 1 min interval. White thrombus was clearly segregated from red blood; and area of the thrombus was calculated.

### Data treatment

The data were treated with Origin 8.1 (OriginLab, USA). Linear regression was used to calculate the inhibition constants. Statistical data treatment gives a coefficient of determination (R^2^) above 0.98 for linearized experimental curves. Curves and statistics for the coagulation tests and the animal experiments were also obtained using Origin 8.1 (OriginLab, USA).

## Results

### Antithrombotic activity of RA-36 aptamer and bivalirudin

The murine model has been applied for comparative study of the RA-36 DNA aptamer and its peptide counterpart, bivalirudin. Doses of RA-36 and bivalirudin were the following. The bivalirudin therapeutic dose is 0.75 mg kg^−1^ (0.34 µmole kg^−1^) for intravenous bolus injection with sequential infusion of 1.75 mg kg^−1^ hr^−1^. We have studied five-fold dose (3.8 mg kg^−1^, 1.7 µmole kg^−1^) and ten-fold dose (7.5 mg kg^−1^, 3.4 µmole kg^−1^) of bivalirudin without subsequent infusions. The therapeutic dose of RA-36 was estimated to be 7 mg kg^−1^ (0.7 µmole kg^−1^) based on our preclinical trials. We have studied two-fold (14 mg kg^−1^, 1.4 µmole kg^−1^), five-fold (35 mg kg^−1^, 3.6 µmole kg^−1^) and ten-fold (70 mg kg^−1^, 7.2 µmole kg^−1^) doses of RA-36 aptamer.

**Figure 1:**
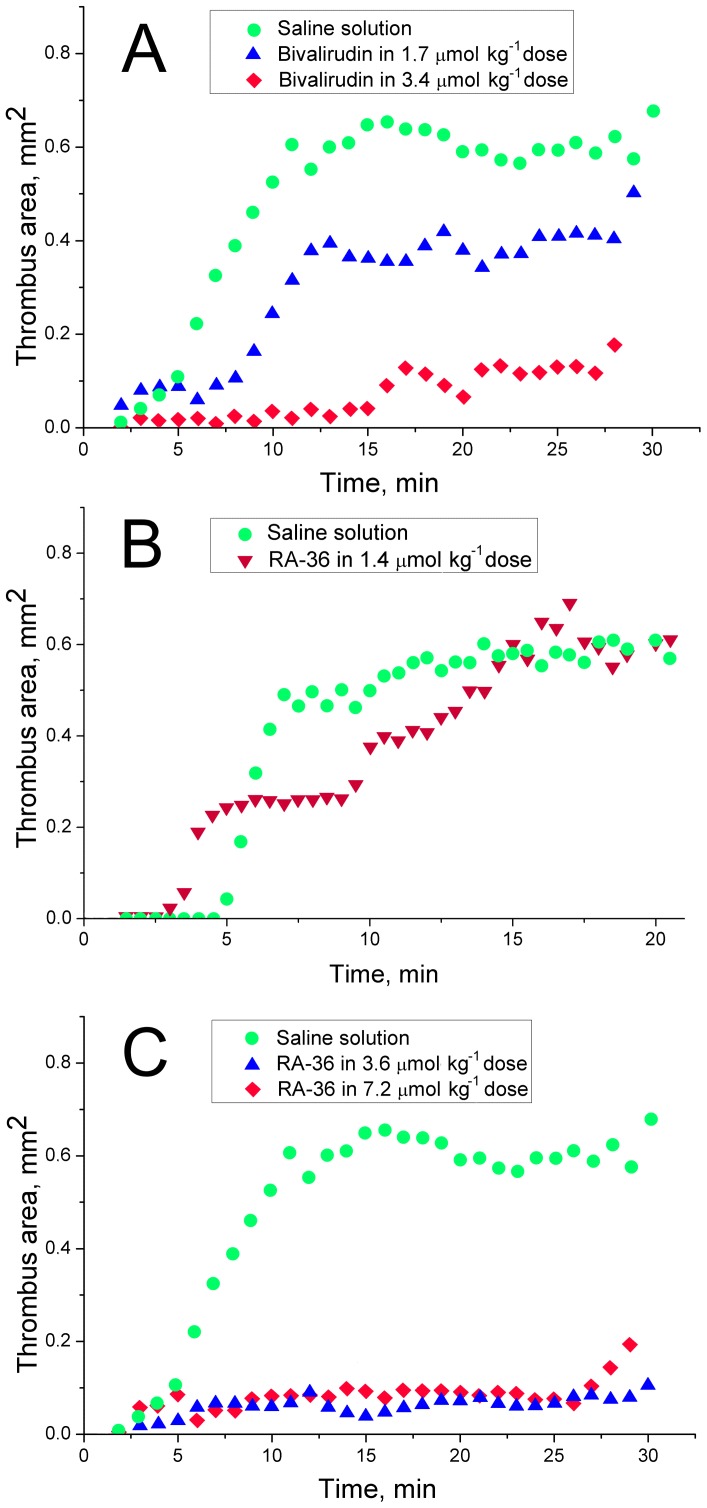
The murine model of thrombosis. The dynamics of thrombus formation at various doses of bivalirudin (A) and RA-36 aptamer (B, C).

The averaged experimental data are depicted in the Figure 1. Not surprisingly, the standard deviations of the data are rather large; the average absolute value of standard deviation is 0.14 mm^2^ (the values vary in the range of 0.05–0.19 mm^2^). The activity-dose dependence is clearly seen for five-fold and ten-fold doses of bivalirudin (Figure 1A). In contrast, five-fold and ten-fold doses of RA-36 aptamer have almost completely prevented formation of thrombus (Figure 1C). Two-fold dose of RA-36 aptamer slightly slows down formation of the thrombus (Figure 1B).

### Inhibition of human and murine thrombins in the enzymatic assay

**Figure 2:**
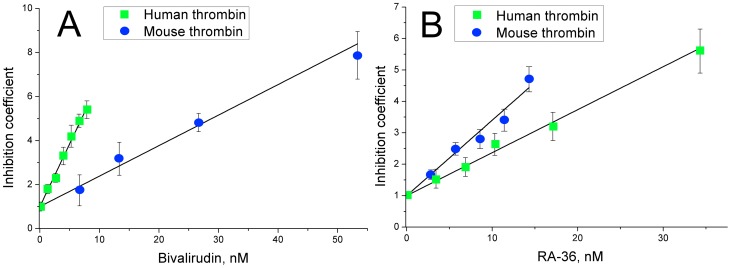
Inhibition of human and murine thrombins in fibrinogen hydrolysis with bivalirudin (A) and RA-36 aptamer (B).

The inhibition curves for human and murine thrombins are compared in the Figure 2. Bivalirudin has pronounced specificity to the human thrombin comparing with the murine thrombin. For bivalirudin the apparent inhibition constants are 1.75±0.04 nM for human thrombin and 7.2±0.3 nM for murine thrombin, respectively. Contrary to bivalirudin, RA-36 aptamer has the apparent inhibition constant for the human thrombin is slightly larger than those for the murine thrombin: 7.5±0.3 nM and 4.4±0.4 nM, respectively (as reported earlier by Zavyalova *et al*. [29]).

### Anticoagulant activity of RA-36 aptamer and bivalirudin

**Figure 3:**
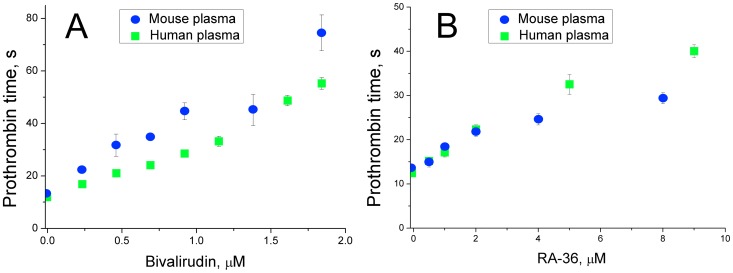
Inhibition of human and murine plasma coagulation with bivalirudin (A) and RA-36 aptamer (B). The coagulation test of prothrombin time was used.

The anticoagulant activity of bivalirudin and RA-36 aptamer was estimated by the coagulation test of prothrombin time (Figure 3). Some difference from the tendencies was revealed. Bivalirudin inhibits the murine blood plasma slightly better than the human one. And RA-36 aptamer inhibits murine and human blood plasma in the equal extent. Bivalirudin anticoagulant activity surpasses RA-36 activity by approximately four-fold.

## Discussion

To study the antithrombotic activity of RA-36 aptamer we have explored the murine model of thrombosis, which is performed by making electric injury of the vessel. And it is the first example of the application of this thrombosis model for the anticoagulant aptamer studying. Previously, two approaches were used to investigate *in vivo* the antithrombotic activity of the aptamers. The first one is based on analysis of the blood samples which are taken from the animal during 1–60 min after bolus or infusion administration of the aptamer. The second approach is based on direct tracking of thrombus formation in aptamer-treated animal.

The first approach was described by DeAnda *et al.* [34]. Mixed breed dogs underwent the cardiopulmonary bypass receiving thrombin aptamer 15-TBA (dGGTTGGTGTGGTTGG) as the anticoagulant. Animals were infused with the aptamer in dose of 0.3–1.5 mg kg^−1^ min^−1^ during 40 min. The blood samples were collected and analyzed with the coagulation tests. The anticoagulant activity of the aptamer was successfully demonstrated. Besides, the thrombosis was monitored visually following a formation of the large thrombi in the extracorporeal blood circle.

Intravenous administration and venous blood sampling was also conducted by Griffin *et al.* [35]. Cynomolgus monkeys received thrombin aptamer 15-TBA (dGGTTGGTGTGGTTGG) in bolus dose of 10 mg kg^−1^ and sequential infusion of 0.3 mg kg^−1^ min^−1^ aptamer during 60 min. The blood samples were collected and analyzed with coagulation tests. Achieving the plateau of the anticoagulant effect was demonstrated.

The analogous experiments were conducted by Diener *et al.* [36] on Sprague-Dawley rats and cynomolgus monkeys. Several thrombin aptamers were bolus administrated intravenously in dose of 1.5–6.4 µmol kg^−1^ with sequential infusion of 0.14–2.5 mg kg^−1^ min^−1^ aptamer. Besides, thrombin aptamer antidote (complementary oligonucleotide) was administrated, and it turned out that the anticoagulant activity of thrombin aptamers was abolished.

The first approach was also used to estimate clearance of thrombin aptamers from the blood of Wistar rats in Zavyalova *et al.* [28] study. Several thrombin aptamers were bolus administrated intravenously in a dose of 0.6–6.2 mg kg^−1^, and the blood samples were collected during 30 min. The anticoagulant effect of the aptamers disappeared in 20 min.

Therefore, analyzing of the blood samples of the aptamer treated animal allows indirect estimating of the anticoagulant activity of aptamers *in vivo*. Aptamer clearance, anticoagulant plateau existence, abolishment of the anticoagulant activity with antidote had been successfully explored. But this approach does not allow direct estimation of the antithrombotic activity of the aptamers.

The second approach is able to gain the complementary data, as it implies *in vivo* investigation of the aptamer effect on the dynamics of thrombus formation within the animal vessel; though there are only few studies. All data had been obtained for the RNA aptamer for von Willebrand factor.

Rusconi *et al.* [24] and Nimjee *et al.* [25] described the antithrombotic activity of aptamer for von Willebrand factor in the murine model thrombosis. Intravenous bolus injection of the aptamer was made in 0.5–3 mg kg^−1^ dose. The transonic flow probe was placed around the carotid artery. Thrombosis was initiated by 10% ferric chloride-soaked piece of paper, which was applied near the flow probe for five minutes. Mean time of thrombosis was 10 min in the control group and up to 60 min in the aptamer treated group.

The thrombosis model with the electric injury of the artery was used by Diener *et al.* [26]. Cynomolgus monkeys were treated with the aptamer in the bolus intravenous dose of 0.1–0.6 mg kg^−1^ and the sequential infusion dose of 1.0–3.7 µg kg^−1^ min^−1^. Doppler flow probe was placed around the carotid artery. The electrode was placed near the flow probe, and electric injury was induced on a par with stenosis of the vessel. The time of full vessel occlusion was measured. The aptamer was shown to increase significantly the time of occlusion.

Besides listed above techniques, there are several thrombosis models that have not been used for antithrombotic aptamer studying. Generally all thrombosis models can be divided into six classes depending on the trigger of thrombosis.

1. **Dissecting the vessel.** Selected vessel is dissected, and the time of bleeding is estimated. One of the simplest techniques is cutting of the tail tip of mouse or rat [37], [38].
2. **FeCl_3_ induction.** A piece of paper wetted in FeCl_3_ solution is imposed around the vessel. Ferric cations damage vessel epithelium inducing thrombosis [39]–[44].
3. **Arteriovenous shunting.** Jugular vein and coronary artery are joined with a plastic tube with a nylon thread inside. The thread promotes thrombosis. Size of the thrombus can be estimated by weighting the thread with adherent thrombus [45]–[49].
4. **Stenosis and stasis.** Vessel stenosis or stasis promotes thrombosis. Other triggers are often used to induce formation of large thrombi [49]–[53].
5. **Phototoxicity.** Fluorescent dye is administrated intravenously. Selected vessel is irradiated with UV waves, and thrombi are formed. Phototoxicity is mediated with reactive oxygen, which is generated through fluorophore excitation. Thrombocytes are the main component of these thrombi; there is just a little amount of fibrin fibers [54], [55].
6. **Electric injury.** Direct current of low amperage (2–250 µA) damages vessel epithelium causing thrombosis. Electric injury can be performed both on veins and arteries [33], [48], [56]–[59].

There are several techniques to estimate a size of the thrombus:

1. **Temperature measurement.** Thrombosis and occlusion of the artery cause significant decrease of the vessel temperature downstream of the thrombus [39], [58].
2. **Time of bleeding.** Mechanical damages, dissections of the vessel cause bleeding which is stopped with formation of a large thrombus [37], [38].
3. **Weighting the thrombus.** The thrombus is cut out, dried and weighted [49]–[52], [59].
4. **Dissecting the vessel.** The vessel with the thrombus is dissected, and area of the white formation, which corresponds to the thrombus, is measured using a microscope [44].
5. **Flow probe.** Blood flow can be estimated with Doppler flow probe. Deceleration of the blood flow indicates the thrombosis [40]–[43], [57].
6. **Video shooting.** Thrombus is clearly separable from bloodstream, because it appears as white plug in the vessel. Video shooting can be used to detect thrombus dynamics in time. Digitizing of the frames allows estimating the thrombus area [33].

To study *in vivo* aptamer activity our murine model of thrombosis involves a combination of electric injury as the trigger of thrombosis and video shooting as the technique for measurement of the thrombus. Advantages of the application of this model to antithrombotic aptamers are as follows: reproducibility of the triggering of the thrombosis, low time of electric injury exposure (1 min versus 5 min in Diener *et al.* [26]), possibility of studying the dynamics of thrombosis, low weight of the mouse as the animal, and therefore very few quantity of the aptamer required.

The murine model has been applied for comparative study of the RA-36 DNA aptamer and its peptide counterpart, bivalirudin. Both RA-36 DNA aptamer and bivalirudin are direct thrombin inhibitors. RA-36 binds with thrombin exosite I blocking fibrinogen binding to the enzyme [30]; whereas bivalirudin binds both with thrombin exosite I and catalytic site, blocking all enzymatic activities of thrombin [31]. Doses of RA-36 and bivalirudin were chosen based on the therapeutic value for bivalirudin and predicted therapeutic value for RA-36 aptamer. We have studied five-fold dose (3.8 mg kg^−1^, 1.7 µmole kg^−1^) and ten-fold dose (7.5 mg kg^−1^, 3.4 µmole kg^−1^) of bivalirudin. And also we have studied two-fold (14 mg kg^−1^, 1.4 µmole kg^−1^), five-fold (35 mg kg^−1^, 3.6 µmole kg^−1^) and ten-fold (70 mg kg^−1^, 7.2 µmole kg^−1^) doses of RA-36 aptamer.

Both bivalirudin and RA-36 were shown to possess the activity-dose dependence (Figure 1). It is worth noting that the thrombus size in two-fold aptamer dose group achieves the thrombus size in control group at fifteenth minute. This result agrees well with the time of full excretion of RA-36 aptamer from the rat blood as it was shown earlier by Zavyalova *et al.* [28]. The time of excretion was estimated to be 15 min at 3.2 mg kg^−1^ (0.3 µmol kg^−1^) dose. In case of bivalirudin obvious excretion-related effect has not been observed in the experiments that correlates with greater half-life of bivalirudin in blood – about 25 min in humans [31].

**Figure 4:**
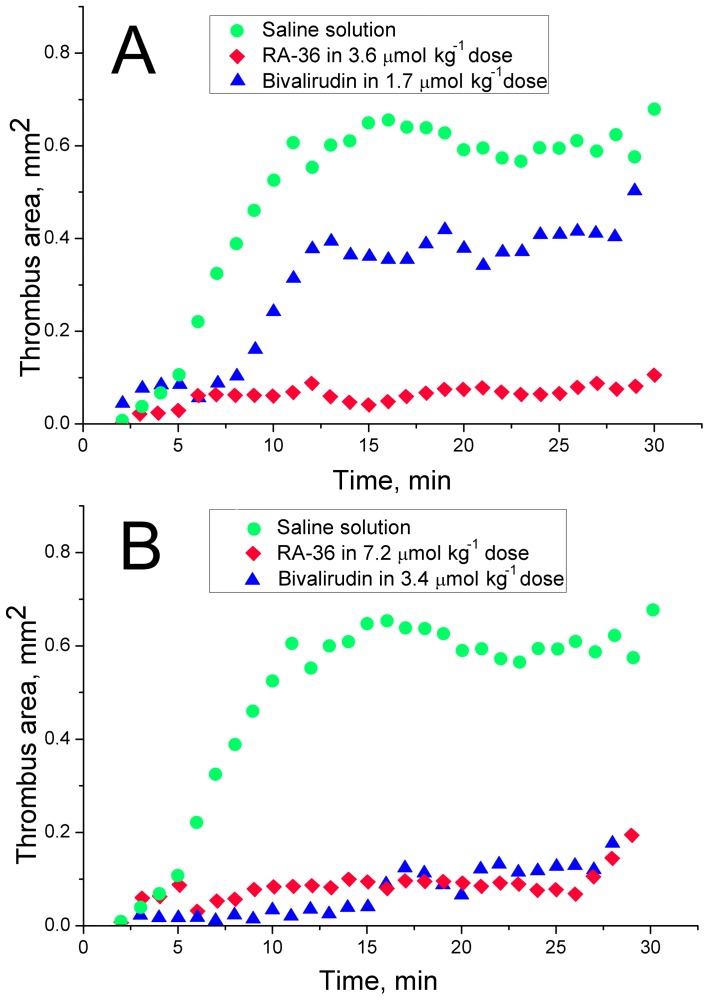
The comparison of the antithrombotic activity of five-fold (A) and ten-fold (B) doses of bivalirudin and RA-36 aptamer.

The antithrombotic activity of bivalirudin and RA-36 aptamer were compared as shown in Figure 4. Five-fold dose of RA-36 aptamer gives much more pronounced antithrombotic effect than five-fold dose of bivalirudin (Figure 4A). Ten-fold dose of each inhibitor has prevented formation of the thrombus similarly; even the data distributions are not significantly different according to nonparametric statistical test (paired sample sign test). Five-fold dose of RA-36 aptamer seems to be equal to ten-fold dose of bivalirudin; statistical treatment also confirms the data distributions to be not significantly different according to nonparametric statistics. Noteworthy, the absolute values of the doses are almost equal – 3.4 µmole kg^−1^ for bivalirudin and 3.6 µmole kg^−1^ for RA-36 aptamer. This result might be due to the different species-specificity of both RA-36 aptamer and bivalirudin. This assumption has been checked in the following experiments.

Earlier the turbidimetric assay has been developed by us to determine the aptamer inhibition constant of thrombin. Briefly, the degree of deceleration of fibrinogen hydrolysis with thrombin is estimated with the ‘inhibition coefficient’. The apparent inhibition constant can be calculated from the slope of the linear dependence of the inhibition coefficient from the inhibitor concentration [32]. We have demonstrated that bivalirudin has pronounced specificity to the human thrombin comparing with the murine thrombin (Figure 2). For bivalirudin the apparent inhibition constants are 1.75±0.04 nM for human thrombin and 7.2±0.3 nM for murine thrombin, respectively. Contrary to bivalirudin, RA-36 aptamer has the apparent inhibition constant for the human thrombin is slightly larger than those for the murine thrombin: 7.5±0.3 nM and 4.4±0.4 nM, respectively (as reported earlier by Zavyalova *et al*. [29]). Therefore the inhibition constants correlate well with *in vivo* antithrombotic efficiency of RA-36 aptamer and bivalirudin in the murine model of thrombosis. So the equal antithrombotic activity of RA-36 aptamer and bivalirudin seems to be a result of the species-specificity of bivalirudin.

The anticoagulant activity of bivalirudin and RA-36 aptamer was estimated by the coagulation test of prothrombin time (Figure 3). Some difference from the tendencies was revealed. Bivalirudin inhibits the murine blood plasma slightly better than the human one. And RA-36 aptamer inhibits murine and human blood plasma in the equal extent. Bivalirudin anticoagulant activity surpasses RA-36 activity by approximately four-fold. Nevertheless the antithrombotic activities of both inhibitors are almost equal in the murine model of thrombosis. Therefore, the antithrombotic activity correlates well with inhibition constants, and less with anticoagulant activity. This might be due to some specific interactions of thrombin-inhibitor complex with other components of blood coagulation cascade.

## Conclusions

The murine model of thrombosis, used in this study, perfectly suits the investigation of novel aptameric antithrombotic agents. The murine thrombin binds G-quadruplex DNA aptamers with efficiency similar to that of the original aptamer target, human thrombin. Comparison of RA-36 aptamer and bivalirudin has revealed that they are the direct thrombin inhibitors of different anticoagulant potency. But species-specificity of the inhibitors leads to the equal physiological effects in the murine model of thrombosis.
